# Exploring PM2.5 Environmental Efficiency and Its Influencing Factors in China

**DOI:** 10.3390/ijerph182212218

**Published:** 2021-11-21

**Authors:** Dongdong Ma, Guifang Li, Feng He

**Affiliations:** 1 School of Economics, Henan University of Economics and Law, Zhengzhou 450046, China; madongdong@huel.edu.cn; 2 Institute of Economics and Management, Henan Agricultural University, Zhengzhou 450046, China; 3 School of Economics and Management, University of Science and Technology Beijing, Beijing 100083, China

**Keywords:** PM2.5 pollution, environmental efficiency, SBM-Undesirable-VRS model, environmental regulations, environmental Kuznets curve, pollution haven

## Abstract

In China, air pollution, especially fine particulate matter (PM2.5) pollution, has become increasingly serious with the rapid economic growth that has occurred over the past 40 years. This paper aims to introduce PM2.5 pollution as a constraint in the environmental efficiency research framework through the use of panel data covering the Chinese provinces from 2001–2018. PM2.5 environmental efficiency is measured with the slack-based measure (SBM)-Undesirable-variable returns-to-scale (VRS) model, and the results show that the average PM2.5 environmental efficiency score is 0.702, which indicates inefficiency, and is U-shaped over time. The PM2.5 environmental efficiency scores are unbalanced across the eight regions and 30 provinces of China. Additionally, the relationship between PM2.5 environmental efficiency and its influencing factors is examined with a tobit model, and the empirical findings indicate that the relationship between economic development and PM2.5 environmental efficiency is an inverted U, which is the opposite of the traditional environmental Kuznets curve (EKC). In addition, technological innovation, trade dependency, and regional development each have a significantly positive effect on PM2.5 environmental efficiency. However, environmental regulations, the industrial structure, and population density have significantly negative effects on PM2.5 environmental efficiency. Finally, this paper fails to prove that foreign direct investment (FDI) has created a PM2.5 “pollution haven” in China.

## 1. Introduction

In China, environmental problems, especially air pollution such as haze pollution, have become increasingly serious with the rapid economic growth (an average of 10% per year) that has occurred since the implementation of China’s economic reform and opening up in 1978 [1,2]. Haze pollution (composed of particulate matter: PM2.5 and PM10) has affected the economy, the transportation systems and the lives of residents in the 30 provinces and major cities in China [3,4]. Mortality due to respiratory and cardiovascular disease has increased because of the continuing outbreak of haze pollution. Statistics show that the annual number of deaths caused by outdoor air pollution in China is conservatively estimated to be between 350,000 and 500,000. The health cost of air pollution has reached 1.16% to 3.8% of GDP. In the 2017 report of the Ninth National People’s Congress, President Xi Jinping stated, “Promoting changes in economic quality, efficiency, and power in order to increase total factor productivity is one of the main paths for transforming China’s economy from having a focus on high-speed growth to a focus on high-quality growth”. Therefore, when measuring environmental efficiency, PM2.5 pollution needs to be included as an important undesired output, as PM2.5 is currently the main environmental pollutant in China. In this paper, environmental efficiency refers to PM2.5 environmental efficiency, which is the measure of environmental efficiency when PM2.5 is included as an undesirable output.

The estimation of environmental efficiency has always been a hot topic in the field of environmental economics, and this has been especially true for Chinese environmental economists since 2008. Many studies have used data envelopment analysis (DEA) to measure environmental efficiency, and these studies have concentrated on the two traditional input variables and economic output [5]. The choice of the two traditional input variables, labour and capital, is relatively restrictive [6,7]. With the growing emphasis on environmental protection and energy conservation, some researchers have begun to use energy consumption as an input variable [8], and renewable and nonrenewable energy consumption were also considered in Apergis et al. [9]. Khoshnevisan et al. [10] also included total water consumption as an input variable. Output is typically divided into desirable output and undesirable output; desirable output is generally defined as economic output and undesirable output is generally defined as environmental pollution. Economic output is typically measured as regional GDP, but the measurement of environmental pollution depends on the choice of environmental pollutants. Studies considering both desirable and undesirable output can be divided into four categories. The first category comprises those studies in which one environmental pollutant, such as sulphur dioxide (SO_2_) [11] or carbon dioxide (CO_2_) [12,13], is selected as the undesirable output to measure the environmental efficiency of different cities in China. The second category includes studies that include two environmental pollutants. Wang et al. [14] put both SO_2_ and CO_2_ into their model for evaluating the environmental efficiency of China’s regions and found that the use of a treatment to transform undesirable outputs into desirable outputs is more appropriate for evaluating China’s regional energy and emissions performance. Among studies in the third category, such as Chen et al. [15] and Zhu et al. [16], the “three wastes”—wastewater, waste gas and solid waste—are often used as undesirable outputs. When calculating environmental efficiency in the fourth category, four undesirable outputs—CO_2_, SOx emissions, NOx emissions and other traditional pollutants—are incorporated in the empirical investigation [17,18]. In addition, some scholars have also measured environmental efficiency and used air pollutants as the undesired outputs. For example, Sueyoshi and Yuan [19] used PM2.5 and PM10 as undesirable outputs when measuring the efficiency of cities in China from 2013 to 2014. Cheng et al. [20] and Guo and Luo [21] estimated environmental efficiency with haze pollution in major Chinese cities as the undesirable output. According to these papers, different undesired outputs lead to different environmental efficiency evaluation results.

In addition, other studies have explored the impact of various factors on environmental efficiency. For instance, Zhang et al. [22] used a tobit regression model to study the factors that influence environmental efficiency. Factors with a positive influence include per capita GDP, population density, capacity for innovation, environmental awareness, and the industrial structure, but energy intensity has a negative influence. Yang and Li [23] examined the impact of various factors on the environmental efficiency of Chinese industry through a regression analysis and found that both FDI and exports had a negative effect on industrial environmental efficiency. Yasmeen et al. [24] found that technological innovation had a positive effect on environmental efficiency at both the national and regional levels in China. Zhong et al. [25] discovered that economic development and energy–environmental efficiency have a U-shaped association, and government regulations and population density have significantly positive effects on energy–environmental efficiency, while the industrial structure and technological progress have negative effects. Yin et al. [26] noted that environmental regulations had a significantly positive effect on environmental efficiency and economic development factors had insignificant positive effects, while technological innovations, the energy consumption structure and the industrial structure had insignificant negative effects. In each of the research studies discussed above, the research area, the research period, and the indicators used are different, resulting in inconsistent estimates of the effects of related factors on environmental efficiency.

Some studies have made valuable attempts to examine environmental efficiency and have built the foundation for this research. Notably, these studies have limitations. First, most of the literature focusing on environmental output considers traditional pollutants, such as SO_2_, CO_2_, and the three wastes [5,6,7,8,9,10,11,12,13,14,15,16]; some scholars have used PM2.5 pollution to examine the environmental efficiency of cities or city groups in China [17,18,19,20,21]. Given this past work, in this paper, PM2.5 environmental efficiency is estimated by using a slack-based measure (SBM)-variable returns-to-scale (VRS) model for the provinces of China. Second, the conclusions regarding the impact of related factors on environmental efficiency in the literature are not consistent [22,23,24,25,26], and there is little research investigating the influence of factors on PM2.5 environmental efficiency, especially whether the relationship between PM2.5 environmental efficiency and economic development generates an environmental Kuznets curve (EKC). Therefore, this paper investigates the existence of an EKC in China. Furthermore, the creation of a “pollution haven” due to foreign direct investment (FDI) and the influence of other factors on PM2.5 environmental efficiency will also be tested with the panel tobit model in the paper.

## 2. Methodology and Methods

### 2.1. Methodology

The first DEA model to deal with undesired outputs was presented by Fǎre et al. [27]. The main approaches to dealing with undesirable outputs include evaluating curve measures, processing pollutants as inputs, evaluating the production function by converting the data, and establishing a distance function. Evaluating curve measures requires nonlinear programming, and the solution is cumbersome; processing pollutants as inputs is not consistent with actual production processes; the evaluating production functions by converting the data is too difficult to be widely used in real life; and establishing distance functions fails to correctly give the direction of the efficiency improvement and is vulnerable to the subjectivity of decision makers. Because of the need for slack in the variables, this paper applies DEA to calculate efficiency scores that account for undesirable outputs. The SBM-Undesirable-VRS model [28] is based on a nonradial and nonoriented model and is shown in Formula (1).

The economy-energy-environment production system has *n* decision making units (DMUs). Every DMU includes three factors: inputs, desirable outputs and undesirable outputs. These factors are represented by three vectors: $x\in R^{m}$, $y^{g}\in R^{s_{2}}$, and $y^{b}\in R^{s_{2}}$. This paper defines the matrices X, $Y^{g}$, and $Y^{b}$ as follows:

$$X=\left[x_{1},x_{2},\cdots ,x_{n}\right]\in R^{m\times n}\;Y^{g}=\left[y_{1}^{g},y_{2}^{g},\cdots ,y_{n}^{g}\right]\in R^{s_{1}\times n}\;Y^{b}=\left[y_{1}^{b},y_{2}^{b},\cdots ,y_{n}^{b}\right]\in R^{s_{2}\times n}$$

Assume that, X>0, $Y^{g}>0$, $Y^{b}>0$. The production technology set (*P*) is defined as follows:

$$P=\{(x,y^{g},y^{b})|x\geq X\lambda ,y^{g}\leq Y^{g}\lambda ,y^{b}\geq Y^{b}\lambda ,\lambda \geq 0\}$$

where λ is the intensity vector, x≥Xλ indicates that the actual input level is greater than that on the frontier, $y^{g}\leq Y^{g}\lambda$ indicates that the actual desirable output level is below that on the frontier, and $y^{b}\geq Y^{b}\lambda$ indicates that the actual undesired output level is higher than that on the frontier. The SBM-Undesirable model can be written as shown in Equation (1):

(1) $$\begin{array}{l} \rho^{*}=\min\frac{1-\frac{1}{m}\sum_{i=1}^{m}\frac{s_{i}^{-}}{x_{i0}}}{1+\frac{1}{s_{1}+s_{2}}(\sum_{r=1}^{s_{1}}\frac{s_{r}^{g}}{y_{r_{0}}^{g}}+\sum_{r=1}^{s_{2}}\frac{s_{r}^{b}}{y_{r0}^{b}})}\\ s.t.\left\{ \begin{array}{l} x_{0}=X\lambda +s^{-}\\ y_{0}^{g}=Y^{g}\lambda -s^{g}\\ y_{0}^{b}=Y^{b}\lambda +s^{b}\\ \lambda \geq 0,s^{-}\geq 0,s^{g}\geq 0,s^{b}\geq 0 \end{array}\right. \end{array}$$

where $s^{-}$ and $s^{b}$ correspond to the excesses in inputs and undesirable outputs, respectively, while $s^{g}$ corresponds to shortages in desirable outputs. The objective function $\rho^{*}$ is strictly decreasing with respect to $s^{-}$, $s^{g}$, and $s^{b}$, and its objective value satisfies ${0<\rho }^{*}\leq 1$.

### 2.2. Data

Four inputs and two outputs were chosen for this paper: the four inputs are employed persons, total energy consumption, the capital stock and total water consumption; the two outputs are GDP (the desirable output) and PM2.5 pollution (the undesirable output). Tibet was not included because the relevant data is missing; therefore, data from 2001–2018 relating to 30 provinces in mainland China were used in this paper. To examine the imbalance in regional development, the research sample was divided into eight regions according to the standard divisions of the National Bureau of Statistics of the People’s Republic of China: Northeast China (NEC; Liaoning, Jilin, and Heilongjiang), Northern Coastal China (NCC; Beijing, Tianjin, Hebei, and Shandong), Eastern Coastal China (ECC; Shanghai, Jiangsu, and Zhejiang), Southern Coastal China (SCC; Fujian, Guangdong, and Hainan), Middle Reaches of the Yellow River (MYR; Shaanxi, Shanxi, Inner Mongolia, and Henan), Middle Reaches of the Yangtze River (MYZR; Hubei, Jiangxi, and Anhui), Southwest China (SWC; Guangxi, Sichuan, Guizhou, and Yunnan), and Northwest China (NWC: Gansu, Qinghai, Ningxia, and Xinjiang).

The first input was employed persons, which is measured as the number of employees at the end of the calendar year. This indicator reflects the actual utilization of all labour resources over a given period of time and is indispensable to the production function [6]. The second input was total energy consumption, which captures the level, composition and growth rate of energy consumption [7]. The capital stock, the third input, is the total existing capital resources in the region. This measure reflects the scale and technical level of existing production and operations [11]. The fourth input was total water consumption, which reflects the total amount of water used in the development and utilization of water resources [8].

Regional GDP [16] and the PM2.5 concentration [29] were selected as proxy indicators for the desirable output and undesirable output, respectively, in this paper.

The data on the number of employed persons, total energy consumption, total water consumption, and GDP were obtained from the City Statistical Yearbooks (2002–2019), the China Energy Statistics Yearbooks (2002–2019) and the China Environmental Statistical Yearbooks (2002–2019). GDP is measured smoothly in constant 2000 prices to eliminate the influence of inflation. The capital stock data were from the results proposed by Ma et al. [4], who assumed a depreciation rate of 10.96% with the perpetual inventory method, and converted them into constant 2000 prices. The formula for calculating the capital stock is given in Equation (2).

(2) $$K_{it}=I_{it}+(1-\delta_{it})K_{it-1}$$

where *K*_it_ is the capital stock of region *i* in year *t*, *I*_it_ is the investment made by region *i* in year *t*, and *δ*_it_ is the depreciation rate for region *i* in year t.

PM2.5 concentration data were estimated from the Atmospheric Composition Analysis Group with aerosol vertical profiles and scattering properties. The dataset on global PM2.5 concentrations has a spatial resolution of 10 km when reporting the annual averages from 2001 to 2018 [30]. The PM2.5 concentration of 30 Chinese provinces from 2001 to 2018 was obtained with ArcGIS 10.0 software (Environmental Systems Research Institute, RedLands, CA, USA). Table 1 provides the statistical descriptions of the input and output variables.

## 3. Measurement of PM2.5 Environmental Efficiency

In this section, MaxDEA6.16 software (MaxDEA Software Ltd., Beijing, China). is used to calculate the PM2.5 environmental efficiency score of 30 Chinese provinces from 2001–2018; the scores are shown in Table 2. Then, the PM2.5 environmental efficiency is analysed separately at both the national and regional level.

### 3.1. Trends in Overall PM2.5 Environmental Efficiency

**Figure 1:**
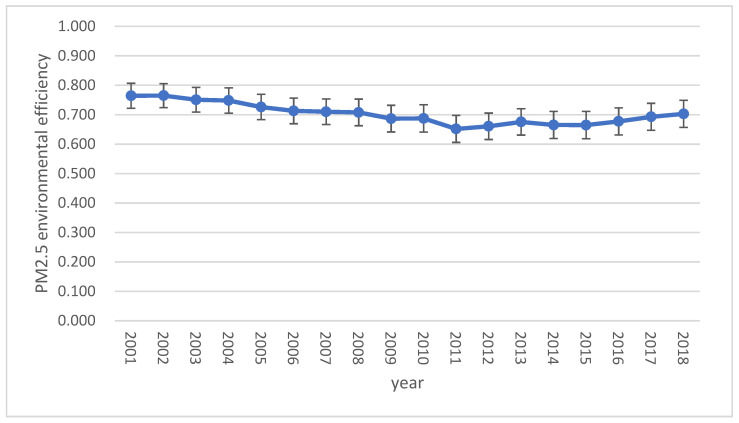
Trend in PM2.5 environmental efficiency in China (2001–2018).

The trend in China’s average PM2.5 environmental efficiency is shown visually in Figure 1, and error bars represent the overall distribution of PM2.5 environmental efficiency. The PM2.5 environmental efficiency level in China is not high, with an average value of only 0.702, and the trend over time is a U-shaped curve. PM2.5 environmental efficiency declined sharply from 2001 to 2011 but slowly improved from 2012 to 2018. Therefore, it is possible that PM2.5 pollution entered into public awareness in 2011 and attracted widespread attention from diverse groups of people, leading to the gradual treatment of PM2.5 pollution and an increase in the PM2.5 environmental efficiency level. Generally, China’s economic development model has resulted in high levels of output and of pollution, and PM2.5 pollution has been ignored. If PM2.5 pollution is addressed, China may be able to obtain economic development with a high yield but low levels of pollution. To achieve high-quality economic growth in the future, it is both necessary and very important to incorporate PM2.5 pollution into China’s environmental efficiency assessment framework.

### 3.2. Differences in PM2.5 Environmental Efficiency

The development of PM2.5 environmental efficiency in the different regions of China has been unbalanced, as shown in Table 2. (1) The highest level of PM2.5 environmental efficiency in China has been achieved in SCC. The average PM2.5 environmental efficiency score for the SCC region is 0.954. In the SCC region, which includes Fujian, Guangdong, and Hainan, tourism and light industry have been given priority. Being the region that developed earlier and that has carried out China’s reform and opening policies since 1978, SCC has developed its own model for economic growth and is affected by PM2.5 pollution only slightly [31]. (2) The PM2.5 environmental efficiency score for the ECC region, which includes Shanghai, Jiangsu, and Zhejiang provinces, is 0.909. ECC is China’s largest economic centre, and its average PM2.5 concentration is 43.33 μg/m^3^. High levels of expected output and low levels of undesirable output cause this region to be ranked second among the eight regions in terms of PM2.5 environmental efficiency. (3) The PM2.5 environmental efficiency score of the NCC region is the third highest. This region includes Beijing, Tianjin, and Hebei and Shandong provinces. The average PM2.5 environmental efficiency score for NCC is 0.882 and has been trending slowly downwards over time. (4) The NEC region, which includes Jilin, Heilongjiang, and Liaoning provinces, is next in terms of environmental efficiency. Its average PM2.5 environmental efficiency score is 0.796, which ranks fourth among the eight regions. NEC belongs to the old industrial base, where the damaged ecological environment has been repaired under governmental environmental regulations, and the average PM2.5 concentration is now only 28.38 μg/m^3^, As a result, the area is moderately environmentally efficient. (5) The NWC region, which includes Gansu, Ningxia, Qinghai, and Xinjiang provinces, ranks fifth in terms of environmental efficiency out of the eight regions. NWC has relatively underdeveloped industry and is accepted as having a manufacturing industry with higher levels of energy consumption than that in the eastern coastal areas. Therefore, the regional governments in NWC should choose high value-added manufacturing industries to avoid heavy haze pollution. (6) The MYR region, which includes Shaanxi, Shanxi, Inner Mongolia, and Henan, ranks sixth in terms of PM2.5 environmental efficiency. In the MYR region, many energy-intensive industries, such as the steel, cement and building materials industries, consume massive amounts of polluting fossil energy, and its average PM2.5 concentration ranks third among the eight regions. All of these industries have caused a decrease in environmental efficiency, especially since 2009. (7) The SWC region, which includes Chongqing, Guangxi, Sichuan, Guizhou, and Yunnan provinces, ranks seventh. There is little industry and few people in the SWC region, so neither its development level nor its pollution level are high, meaning that its environmental efficiency score is not high either. (8) The MYZR region has the lowest environmental efficiency score. The MYZR area, which includes Anhui, Hubei, Hunan, and Jiangxi provinces, is a developing region in China that follows the traditional “pollute and then treat” model. These results all indicate that decision makers should pursue long-term goals and move away from the traditional development model to ensure sustainable development.

**Figure 2:**
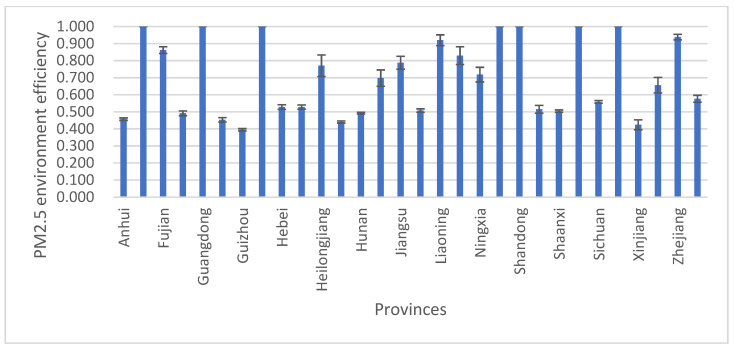
The average environmental efficiency score for each province.

The PM2.5 environmental efficiency scores are also very different across provinces, as shown in Table 3 and Figure 2. (1) The time-series data in Table 3 show that there were 14 provinces with PM2.5 environmental efficiency scores on the frontier of production in 2001., Those 14 provinces are Beijing, Tianjin, Fujian, Guangdong, Hainan, Liaoning, Shandong, Zhejiang, Shanghai, Heilongjiang, Jilin, Inner Mongolia, Ningxia and Qinghai. In 2018, there were 10 provinces with PM2.5 environmental efficiency scores on the frontier of production: Beijing, Tianjin, Guangdong, Hainan, Shandong, Shanghai, Qinghai, Jiangsu, Inner Mongolia and Zhejiang. The number of DEA-efficient provinces fell by 28.57%, which may have been due to changes in PM2.5 pollution during the research period. (2) The cross-sectional perspective in Figure 2 shows that seven provinces—Beijing, Tianjin, Guangdong, Hainan, Shandong, Shanghai, and Qinghai—were on the frontier of production in all years; they were the most efficient of all 30 provinces. Among these seven provinces, three provinces are in NCC, Shanghai is in ECC, two provinces are in SCC, and Qinghai is in NWC. Figure 2 also shows that the remaining 23 provinces—Zhejiang, Liaoning, Fujian, Inner Mongolia, Jiangsu, Heilongjiang, Ningxia, Jilin, Yunnan, Chongqing, Sichuan, Hebei, Henan, Shanxi, Jiangxi, Shaanxi, Hunan, Gansu, Anhui, Guangxi, Hubei, Xinjiang and Guizhou—have low levels of PM2.5 environmental efficiency. One possible reason for this result is that these provinces have serious PM2.5 pollution and many energy-intensive industries, such as the steel, cement, and building materials industries, which consume large amounts of polluting fossil energy.

## 4. Factors Influencing PM2.5 Environmental Efficiency

To better explain the differences in PM2.5 environmental efficiency among areas in China, improve the quality of China’s economic growth and provide complete information for decision-making about the control of PM2.5 pollution, the main factors that influence PM2.5 environmental efficiency in China, the mechanisms underlying their influence, and their actual effects need to be discussed. The EKC and the PM2.5 pollution haven hypothesis also need to be tested against China’s experience. These tests are conducted in this section.

### 4.1. Econometric Model

In environmental economics, the EKC is usually used to examine the relationship between PM2.5 pollution and economic development. Grossman and Krueger [32] found an inverted U-shaped relationship between pollution emissions and national income levels with an econometric model. Based on the EKC model, the panel data tobit model in Equation (3) can be established:

(3) $$\begin{array}{l} ln{EF}_{it}=a_{i}+\beta_{1}ln{PGDP}_{it}{+\beta }_{2}{\left(ln{PGDP}_{it}\right)}^{2}+\beta_{3}{lnSGDP}_{it}+\\ \beta_{4}ln{POP}_{it}+\beta_{5}D_{it}+\beta_{6}{lnTRADE}_{it}+\beta_{7}{lnFDI}_{it}+\\ \beta_{8}ln{TECH}_{it}+\beta_{9}{R\&D}_{it}+\beta_{10}ln{ENVR}_{it}+\varepsilon_{it} \end{array}$$

where *i* and *t* refer to the province and the year, respectively. *EF* represents PM2.5 environmental efficiency, ε is the error term, and the *β* terms are the estimated parameters. PGDP represents the level of economic development, and its square term is also included in the tobit model. This model can be used to describe the relationship between environmental efficiency and economic development. In addition, the industrial structure, regional factors, the degree of openness, technological innovation, and environmental regulations also affect PM2.5 environmental efficiency. By drawing on the existing literature and related experience, some predictions about the sign of the variable’s influence on the dependent variable are made. The definitions and symbols of these variables, as well as their predicted signs, are shown in Table 4.

*SGDP* refers to the ratio of the value added in the secondary industry to GDP. Normally, the secondary industry is dominated by heavy industry; the higher the ratio of the value added in the secondary industry to GDP is, the greater the industrial pollution and the lower the PM2.5 environmental efficiency score. Its expected sign is negative.

Regional factors reflect the degree to which the characteristics of the different regions influence PM2.5 environmental efficiency. Here, *POP* and *D* are chosen as the proxy indicators. *POP* is the provincial population density at the end of the year, calculated as population/square kilometres. Cropper and Griffiths [33] argued that the higher the population density is, the greater is the pressure on the surrounding environment, so that a higher density degrades the environment. However, Selden and Song [34] confirmed that countries with a high population density pay more attention to the environment; therefore, density enhances people’s awareness and the intensity of environmental protection. *D* is a regional dummy variable; if a province is in the eastern region, D = 1, and if not, D = 0. (According to the National Bureau of Statistics, the provinces in the Eastern region include Beijing, Tianjin, Hebei, Liaoning, Shanghai, Jiangsu, Zhejiang, Fujian, Shandong, Guangdong, Guangxi, and Hainan).

The degree of openness can be regarded as measuring China’s progress in entering the world economy. TRADE represents the ratio of total imports and exports to regional GDP [22]. It is used to assess the impact of the development of regional trade on haze pollution. Do relatively high environmental quality standards in developed countries tend to improve the quality of the Chinese environment, or do they lead to more serious environmental pressure? The answer to this question needs to be further verified. FDI is the ratio of FDI to regional GDP [23] and is used to test the applicability of the pollution haven hypothesis to the Chinese experience. On the one hand, FDI introduces advanced technology into the host country. On the other hand, FDI may also shift polluting industries into that country. Hence, the impact of FDI on PM2.5 environmental efficiency is uncertain.

Technological innovation is a key factor influencing energy demand, climate change and environmental governance [24]. There are no standard measurable indicators for technological innovation because technological innovation is a complex and diverse process. To comprehensively evaluate the impact of technological innovation on PM2.5 environmental efficiency, proxy indices related to technological innovation inputs and outputs are selected. R&D is chosen as the proxy indicator for inputs. R&D is the ratio of R&D expenditures to regional GDP. Additionally, TECH is chosen as the proxy indicator for outputs. TECH is the number of patents granted in a region. Advanced technological innovation helps improve environmental indicators and control PM2.5 pollution, so its coefficients are expected to be positive.

ENVR refers to the ratio of total environmental investments to regional GDP [25,26]. It represents the amount of attention given to environmental pollution control across regions. Strict environmental regulations show that the government has made the environment a greater priority, and enterprises with higher environmental standards emit fewer pollutants. However, production costs and burdens increase, which hinders the growth in economic output. In this case, it is difficult to determine the effect of either strict or loose environmental regulations on PM2.5 environmental efficiency.

### 4.2. Data Sources and Descriptive Statistics

The annual data for PGDP, SGDP, ENVR, TRADE, FDI, R&D, TECH, and POP were obtained from the China Energy Statistics Yearbooks (2002–2019), China Environmental Statistical Yearbooks (2002–2019), China Statistical Yearbooks (2002–2019), and China Foreign Trade Statistical Yearbooks (2002–2019). GDP is measured smoothly in constant 2000 prices. Total foreign trade is converted into RMB with the exchange rates in the statistical yearbooks. Table 5 presents the descriptive statistics of the variables.

### 4.3. Analysis of Results

To determine the stability of each coefficient, the variables used in the tobit model are entered one by one into Stata 12.0 (StataCorp LP, Lakeway, USA). Equation (3) is estimated via regression, and the results are shown in Table 6 (see columns 1–6).

According to the results of these seven regressions, there is no significant fluctuation in the size of the coefficients on the independent variables, and there is no change in the sign of the coefficients. Therefore, the influence of each variable on the dependent variable is relatively robust.

(1) Verification of the EKC for PM2.5 environmental efficiency in China. The main effect of PGDP is positive, and its square is negative; both coefficients are significant at the 1% level (see columns 1–6 in Table 6). There is an inverted U-shaped relation between environmental efficiency and economic development. The traditional EKC indicates that environmental pollution first rises and then decreases as the level of economic development increases. However, the inverted U-shaped curve is precisely the opposite of the traditional EKC. This estimate suggests that the increasing the level of economic development increases demand for a high-quality surrounding environment before the turning point, which increases the pressure on the government to control PM2.5 pollution and improve PM2.5 environmental efficiency. However, the opportunity cost of environmental regulation increases when per capita GDP passes the turning point, increasing the difficulty of reducing pollutant emissions so that economic development is no longer an important factor in improving PM2.5 environmental efficiency.

(2) Industrial structure. The coefficient on SGDP is significantly negative, which means that the ratio of value added in the secondary industry to regional GDP has a significantly negative effect on PM2.5 environmental efficiency (see columns 1–6 in Table 6). In China, this ratio was 45.9% in 2001 and 40.7% in 2018. Although the index declined slightly, its base is still high. Industrial enterprises still consume large amounts of energy and resources and discharge a large number of environmental pollutants, meaning that PM2.5 environmental efficiency is restricted.

(3) Regional factors. Two indicators measure the regional characteristics, with contrasting results. The coefficient on POP is significantly negative, but the coefficient on *D* is significantly positive (see columns 1–6 in Table 6). These results are not consistent with those of Song et al. [8]; they suggest that population density has a significantly negative relationship with PM2.5 environmental efficiency. The environmental pressure brought about by increases in population density is greater than the positive effect of increased population density. An increase in population density increases the difficulty of protecting the natural environment, which worsens PM2.5 pollution and reduces PM2.5 environmental efficiency [34]. The dummy variable for the eastern region significantly improves the PM2.5 environmental efficiency score, which shows that the eastern region, with its advanced economic development, has advantages in terms of capital, talent and the establishment of firms and can help improve PM2.5 environmental efficiency.

(4) Degree of openness degree. The coefficient on TRADE is significantly positive, which means that the ratio of total imports and exports to regional GDP has a significantly positive effect on environmental efficiency (see columns 2–6 in Table 6). On the one hand, this may be because foreign requirements with high standards can force improvements in PM2.5 environmental efficiency; on the other hand, foreign trade can improve residents’ living standards and income while increasing their demand for environmental quality, resulting in the control of environmental and PM2.5 pollution and the improvement of PM2.5 environmental efficiency.

The coefficient on *FDI* indicates a negative effect on PM2.5 environmental efficiency (see columns 3–6 in Table 6), but is not significant, which means that there is no evidence to support the hypothesis that China is a pollution haven. Due to factors such as the relative prices of intermediate goods, the level of bilateral trade, market size and other factors, FDI brings many manufacturing firms that consume large amounts of energy or firms in other pollution-intensive industries to China, leading to the accumulation of pollution, especially PM2.5 pollution, in the environment. However, FDI has also introduced advanced technologies and rich experiences, both of which have reduced pollution emissions to a certain extent. Therefore, the impact of FDI on China’s PM2.5 pollution is uncertain.

(5) Technological innovation. The coefficients on *TECH* and R&D are significantly positive, which means that the ratio of R&D expenditures to regional GDP and the number of patents granted in each region have a significantly positive effect on PM2.5 environmental efficiency (see columns 4–6 in Table 6). As an input into technological innovation, R&D expenditure was 104.25 billion RMB in 2001 and 19.67422 trillion RMB in 2018, accounting for 0.95% and 2.16% of GDP, respectively. The 9% annual growth rate shows that China has increased its emphasis on science and technology, which can provide effective financial support for PM2.5 pollution control. As an output of technological innovation, the average number of patents granted in each region was 99,278 in 2001 and 1802,166 in 2018; the average annual increase was 94,604—an amazing rate of growth—which has led to good technological support for PM2.5 control. However, the number of patents granted varies greatly across regions; the average extreme difference was 332,582 between 2001 and 2018. Therefore, the developed eastern areas should provide technological support for the less developed central and western regions in order to narrow the technological gap among regions and improve PM2.5 environmental efficiency.

(6) Environmental regulation. The coefficient on ENVR is significantly negative, which means that the ratio of total environmental investments to regional GDP has a significantly negative effect on PM2.5 environmental efficiency (see column 7 in Table 6). This situation may be related to the model that China uses for environmental pollution control, which is based on end-of-pipe treatment. On the one hand, the absolute value of environmental pollution control investments was 101.5 billion RMB in 2001 and 546.8 billion RMB in 2018, with an average increase of 24.74 billion RMB per year. However, the increase in environmental pollution control is due to the increase in pollutants; the more pollutants there are, the higher the investment in governance, and there is currently no effective control for PM2.5 pollution. On the other hand, the ratio of total environmental investments to regional GDP was only 1.32% during our study period: the maximum value was only 4.24%, and the minimum value was 0.05%, which could indicate that China has not paid enough attention to the environment and PM2.5 pollution and that investments in environmental control are not sustainable. Therefore, current environmental regulation policy in China needs to be further improved, and the implementation of policies directed at the sources of pollution could solve the fundamental environmental and PM2.5 pollution problems.

### 4.4. Robustness Test

To test the robustness of the results, the study period was shortened to 2001–2012 and the model was re-estimated. The results are shown in Table 7. The regression results in Table 7 show that the sign and significance of each variable have not changed significantly, which reveals that the conclusions are robust.

## 5. Conclusions and Policy Implications

With the SBM-Undesirable-VRS model, panel data on Chinese provinces from 2001–2018 were used to measure PM2.5 environmental efficiency. Then, the EKC and other influencing factors in China were examined by using the tobit model. The crucial findings are as follows. The national PM2.5 environmental efficiency score indicates inefficiency, and the average environmental efficiency score is 0.702 and exhibits a U-shape over time. The development of PM2.5 environmental efficiency is unbalanced across the eight regions and 30 provinces of China. Moreover, there is an inverted U-shaped relationship between economic development and PM2.5 environmental efficiency, which is in contrast to the traditional EKC. Technological innovation, trade dependency, and regional development all have a significantly positive impact on PM2.5 environmental efficiency. However, environmental regulations, the industrial structure, and population density can significantly inhibit improvements in PM2.5 environmental efficiency. Finally, FDI has a negative effect on PM2.5 environmental efficiency, but its coefficient is not significant.

The conclusions of this study suggest some policy implications. First, to improve the PM2.5 environmental efficiency of the whole country, it is key that regional emissions standards and regional coordination mechanisms, such as charging different pollution emission taxes according to the degree of economic development in the various regions, be established, as this would be conducive to controlling PM2.5 pollution and narrowing the gap in regional PM2.5 environmental efficiency. The inefficient regions can become efficient by adjusting their inputs and outputs to reach benchmark levels. Second, China should transform its traditional model of economic development, which is characterized by high levels of input, energy consumption, pollution and emissions, into a model of ecological development with high levels of output and low levels of energy consumption, pollution and emissions. This model would account for both economic growth and the quality of that growth together. Third, the government should establish and improve relevant policies and environmental standards for PM2.5 governance. The government should also establish market mechanisms, including emissions trading, pollution permits, and ecological offset mechanisms. To achieve the goals of PM2.5 governance and improvements in PM2.5 environmental efficiency, the end-of-pipe model should be replaced by a focus on pollution prevention and process control, obsolete equipment should be eliminated, and new technologies should be introduced to reduce water and fossil fuel consumption. Fourth, regarding technological innovation inputs, regional governments should guide enterprises to expand their financing channels, strengthen their R&D investments in PM2.5 control technology, and protect the sources of technological innovation. Regarding technological innovation outputs, regional governments should encourage enterprises to actively develop appropriate patents or PM2.5 governance technology to control PM2.5 pollution and improve regional PM2.5 environmental efficiency based on China’s environmental standards. Fifth, the industrial structure should be further optimized, the share of heavy industry should be reduced, and green manufacturing should be actively developed to enable PM2.5 governance and improvements in PM2.5 environmental efficiency. The government should pay more attention to the introduction of advanced technology and of management experience to prevent China from becoming a pollution haven.

## Figures and Tables

**Table 1:** Statistical descriptions of the variables.

	The Main Variables	Units	Average	Median	Min	Max	Stedv.
Input	Employed Persons	10,000 Persons	2509.92	2067.65	268	6767	1679.60
	Total Energy Consumption	10,000 tce	11,377.28	9179.01	520	38,723	7891.53
	Capital Stock	100 million yuan	24,568.7	16,611.35	745.25	138,711.80	24,595.78
	Total Water Consumption	100 million cu.m	195.64	182.7	19.94	591.3	138.58
Desired output	GDP	100 million yuan	10,241.34	7207.65	295.42	62,039.21	10,095.90
Undesired output	PM2.5 concentration	μg/m^3^	34.86	32.42	5.91	85.96	17.83

**Table 2:** PM2.5 environmental efficiency values across the eight regions (2001–2018).

Year	NEC	NCC	ECC	SCC	MYR	MYZR	SWC	NWC
2001	1.000	0.902	0.892	1.000	0.672	0.508	0.537	0.774
2002	0.954	0.902	0.900	1.000	0.700	0.525	0.534	0.773
2003	0.923	0.897	0.884	1.000	0.684	0.505	0.526	0.764
2004	0.970	0.899	0.893	0.969	0.689	0.491	0.497	0.776
2005	1.000	0.898	0.857	0.940	0.659	0.474	0.506	0.682
2006	0.970	0.893	0.846	0.940	0.645	0.458	0.487	0.672
2007	0.973	0.892	0.831	0.924	0.641	0.457	0.494	0.667
2008	0.897	0.887	0.837	0.930	0.618	0.435	0.476	0.764
2009	0.903	0.885	0.827	0.935	0.599	0.431	0.467	0.639
2010	0.935	0.884	0.836	0.945	0.590	0.424	0.443	0.647
2011	0.650	0.872	0.925	0.924	0.528	0.463	0.438	0.584
2012	0.659	0.872	0.936	0.936	0.518	0.469	0.468	0.586
2013	0.656	0.871	0.975	0.944	0.524	0.478	0.510	0.593
2014	0.538	0.862	0.947	0.938	0.502	0.440	0.610	0.599
2015	0.530	0.863	0.972	0.944	0.487	0.446	0.596	0.584
2016	0.554	0.866	1.000	0.949	0.504	0.478	0.600	0.577
2017	0.570	0.868	1.000	1.000	0.535	0.479	0.607	0.587
2018	0.646	0.865	1.000	0.955	0.595	0.463	0.484	0.583
Mean	0.796	0.882	0.909	0.954	0.594	0.468	0.515	0.658
SD	0.186	0.015	0.063	0.027	0.073	0.028	0.055	0.079
SEM	0.044	0.003	0.015	0.006	0.017	0.007	0.013	0.019

Note: SD is the abbreviation of standard deviation, which can reflect the degree of dispersion of a data set; SEM is the abbreviation of the standard error of the mean, which describe the degree of dispersion of the sample mean from the overall expected value. SD and SEM in the following have the same meaning as here.

**Table 3:** PM2.5 Environmental efficiency scores for the 30 Chinese provinces in specific years.

Province	2001	2005	2010	2015	2018	Mean	SD	SEM
Anhui	0.487	0.486	0.407	0.439	0.430	0.450	0.032	0.014
Beijing	1.000	1.000	1.000	1.000	1.000	1.000	0.000	0.000
Fujian	1.000	0.821	0.836	0.831	0.866	0.871	0.066	0.030
Gansu	0.511	0.567	0.534	0.431	0.459	0.501	0.049	0.022
Guangdong	1.000	1.000	1.000	1.000	1.000	1.000	0.000	0.000
Guangxi	0.529	0.481	0.369	0.420	0.421	0.444	0.055	0.025
Guizhou	0.371	0.374	0.384	0.419	0.418	0.393	0.021	0.010
Hainan	1.000	1.000	1.000	1.000	1.000	1.000	0.000	0.000
Hebei	0.608	0.591	0.538	0.452	0.460	0.530	0.064	0.029
Heilongjiang	0.554	0.509	0.440	0.534	0.452	0.498	0.045	0.020
Henan	1.000	1.000	1.000	0.441	0.630	0.814	0.235	0.105
Hubei	0.434	0.405	0.402	0.438	0.445	0.425	0.018	0.008
Hunan	0.536	0.488	0.474	0.494	0.497	0.498	0.021	0.009
InnerMongolia	1.000	1.000	0.804	0.474	0.581	0.772	0.214	0.096
Jiangsu	0.675	0.655	0.664	1.000	1.000	0.799	0.164	0.074
Jilin	0.604	0.532	0.462	0.462	0.514	0.515	0.059	0.026
Jinagxi	1.000	1.000	1.000	0.675	0.725	0.880	0.165	0.074
Liaoning	1.000	1.000	1.000	0.524	1.000	0.905	0.213	0.095
Ningxia	1.000	0.606	0.580	0.614	0.599	0.680	0.179	0.080
Qinghai	1.000	1.000	1.000	1.000	1.000	1.000	0.000	0.000
Shaanxi	1.000	1.000	1.000	1.000	1.000	1.000	0.000	0.000
Shandong	0.605	0.642	0.446	0.413	0.448	0.511	0.105	0.047
Shanghai	0.530	0.486	0.472	0.477	0.480	0.489	0.023	0.011
Shanxi	1.000	1.000	1.000	1.000	1.000	1.000	0.000	0.000
Sichuan	0.618	0.597	0.543	0.545	0.546	0.570	0.035	0.016
Tianjin	1.000	1.000	1.000	1.000	1.000	1.000	0.000	0.000
Xinjiang	0.585	0.553	0.472	0.289	0.276	0.435	0.145	0.065
Yunnan	0.630	0.571	0.477	1.000	0.552	0.646	0.205	0.092
Zhejiang	1.000	0.916	0.845	0.915	1.000	0.935	0.066	0.029
Chongqing	0.653	0.500	0.467	0.650	0.698	0.594	0.103	0.046

Data source: Computed with MaxDEA6.16 software using the SBM-Undesirable-VRS model.

**Table 4:** Influencing factors and their relevant symbols.

Explanatory Variable	Variable Symbol	Variables’ Definition	Prediction
Economy	PGDP	GDP per capita	EKC
Industrial structure	SGDP	The ratio of value added in the secondary industry to regional GDP	-
Regional factors	POP	The ratio of the total population to the regional area at the end of the year	?
	D	Eastern province; D = 1 if yes, and if not, D = 0	+
Openness degree	TRADE	The ratio of total imports and exports to regional GDP	?
	FDI	The ratio of FDI to regional GDP	?
Technology innovation	R&D	The ratio of R&D expenditures to regional GDP	+
	TECH	The number of patents granted in the region	+
Environmental regulation	ENVR	The ratio of total environmental investment to regional GDP	?

Note: “-” means that the variable is predicted to have a negative influence. “+” means that the variable is predicted to have a positive influence. “?” indicates that the influence of the variable is uncertain.

**Table 5:** Descriptive statistics of the variables.

Variables	Mean	Min	Max	Std.dev
PGDP (RMB)	27,709.08	3001.86	155,178.16	25,254.62
SGDP (%)	45.22	16.54	59.05	7.07
ENVR (%)	1.32	0.05	4.24	0.68
TRADE (%)	30.88	1.70	172.15	37.85
FDI (%)	2.64	0.01	9.52	2.06
TECH (Pieces)	48,353.81	124	793,819	92,615.78
R&D (%)	1.34	0.14	6.01	1.05
POP (person/km^2^)	388.47	6.01	3825.69	534.96

**Table 6:** Estimation results for tobit model.

	(1)	(2)	(3)	(4)	(5)	(6)
a	−2.165 (−0.82)	−2.167 (−0.83)	−2.199 (−0.83)	−2.931 (−1.12)	−4.059 (−1.52)	−4.351 (−1.63)
LNPGDP	0.717 (1.73 *)	0.718 (1.72 *)	0.724 (1.70 *)	0.950 (1.80 *)	1.200 (2.21 **)	1.226 (2.34 **)
(LNPGDP)^2^	−0.045 (−1.71 *)	−0.047 (−1.69 *)	−0.047 (−1.70 ***)	−0.068 (−2.39 **)	−0.082 (−2.21 ***)	−0.085 (−2.90 ***)
LNSGDP	−0.345 (−2.40 **)	−0.333 (−2.30 **)	−0.328 (−2.20 **)	−0.270 (−1.85 *)	−0.247 (−1.71 *)	−0.241 (−1.68 *)
LNPOP	−0.228 (−2.69 ***)	−0.216 (−2.77 ***)	−0.216 (−2.75 ***)	−0.068 (−3.05 ***)	−0.284 (−3.05 ***)	−0.294 (−3.06 ***)
D	1.19 (6.07 ***)	1.200 (5.99 ***)	1.202 (5.95 **)	1.381 (6.42 *)	1.359 (6.41 ***)	1.366 (6.38 ***)
LNTRADE		0.069 (2.09 **)	0.070 (2.08 **)	0.071 (2.12 **)	0.070 (2.09 **)	0.066 (1.98 **)
LNFDI			−0.004 (−0.14)	−0.020 (−0.82)	−0.021 (−0.87)	−0.019 (−0.78)
LNTECH				0.087 (2.80 ***)	0.081 (2.62 ***)	0.085 (2.73 ***)
R&D					0.052 (1.81*)	0.056 (2.73 **)
LNENVR						−0.057 (−1.70 *)
Sigma_u	0.400 (5.92 ***)	0.401 (5.96 ***)	0.403 (5.88 ***)	0.474 (5.74 ***)	0.465 (5.72 ***)	0.465 (5.72 ***)
Obs	360	360	360	360	360	360
Log Likelihood	54.235	54.230	54.240	57.912	60.830	60.830

Note: ***, **, * indicate significance at the 1%, 5%, and 10% levels, respectively; the values in parentheses are z values.

**Table 7:** Robustness test results.

Variables	Results	Variables	Results
a	−7.543	LNFDI	−0.813
	(−3.15 **)		(−0.75)
LNPGDP	2.191	LNTECH	0.072
	(4.65 ***)		(2.75 **)
(LNPGDP)2	−0.135	R&D	0.042
	(−5.25 ***)		(1.73 *)
LNSGDP	−0.778	LNENVR	−0.05
	(−2.63 **)		(−2.55 **)
LNPOP	−0.381	Sigma_u	0.536
	(−2.99 **)		(5.06 ***)
D	1.498	Obs	360
	(6.06 ***)	Log Likelihood	97.82
LNTRADE	0.235		
	(2.40 **)		

Note: ***, **, * indicate significance at the 1%, 5%, and 10% levels, respectively; the values in parentheses are z values.

## Data Availability

Data supporting the conclusions of this article are included within the article. The dataset generated and/or analyzed during the present study is available from the corresponding author.
